# Active Tuberculosis Is Associated with Worse Clinical Outcomes in HIV-Infected African Patients on Antiretroviral Therapy

**DOI:** 10.1371/journal.pone.0053022

**Published:** 2013-01-02

**Authors:** Abraham M. Siika, Constantin T. Yiannoutsos, Kara K. Wools-Kaloustian, Beverly S. Musick, Ann W. Mwangi, Lameck O. Diero, Sylvester N. Kimaiyo, William M. Tierney, Jane E. Carter

**Affiliations:** 1 School of Medicine, College of Health Sciences, Moi University, Eldoret, Kenya; 2 USAID-Academic Model Providing Access to Healthcare (AMPATH) Partnership, Eldoret, Kenya; 3 Indiana University School of Medicine, Indianapolis, Indiana, United States of America; 4 Warren Alpert Medical School of Brown University, Providence, Rhode Island, United States of America; 5 Regenstrief Institute, Inc., Indianapolis, Indiana, United States of America; Institut National de la Santé et de la Recherche Médicale, France

## Abstract

**Objective:**

This cohort study utilized data from a large HIV treatment program in western Kenya to describe the impact of active tuberculosis (TB) on clinical outcomes among African patients on antiretroviral therapy (ART).

**Design:**

We included all patients initiating ART between March 2004 and November 2007. Clinical (signs and symptoms), radiological (chest radiographs) and laboratory (mycobacterial smears, culture and tissue histology) criteria were used to record the diagnosis of TB disease in the program’s electronic medical record system.

**Methods:**

We assessed the impact of TB disease on mortality, loss to follow-up (LTFU) and incident AIDS-defining events (ADEs) through Cox models and CD4 cell and weight response to ART by non-linear mixed models.

**Results:**

We studied 21,242 patients initiating ART–5,186 (24%) with TB; 62% female; median age 37 years. There were proportionately more men in the active TB (46%) than in the non-TB (35%) group. Adjusting for baseline HIV-disease severity, TB patients were more likely to die (hazard ratio – HR = 1.32, 95% CI 1.18–1.47) or have incident ADEs (HR = 1.31, 95% CI: 1.19–1.45). They had lower median CD4 cell counts (77 versus 109), weight (52.5 versus 55.0 kg) and higher ADE risk at baseline (CD4-adjusted odds ratio = 1.55, 95% CI: 1.31–1.85). ART adherence was similarly good in both groups. Adjusting for gender and baseline CD4 cell count, TB patients experienced virtually identical rise in CD4 counts after ART initiation as those without. However, the overall CD4 count at one year was lower among patients with TB (251 versus 269 cells/µl).

**Conclusions:**

Clinically detected TB disease is associated with greater mortality and morbidity despite salutary response to ART. Data suggest that identifying HIV patients co-infected with TB earlier in the HIV-disease trajectory may not fully address TB-related morbidity and mortality.

## Introduction

The global burden of tuberculosis (TB) and HIV co-infections is immense. Of the 8.7 million incident cases of TB in 2011, an estimated 1.13 million (13%) were infected with HIV, of whom 430,000 (38%) died. [1] The highest rates of HIV co-infection were reported for TB patients in the African Region where 46% of those with a HIV test were HIV-positive. In some countries in the region, this figure was as high as 70%. [1]. Africa is responsible for 79% of the HIV/TB infections globally. [1], [2] In Kenya, 106,000 cases of TB were registered in 2010 with 41% being HIV co-infected [3].

HIV and TB form a lethal combination, each disease fuelling the other. HIV infection is the most significant known risk factor for acquisition of TB infection and development of TB disease [4], [5] while TB accelerates HIV disease progression. [6]–[9] TB is undoubtedly the leading cause of death in the setting of AIDS, accounting for about 26% of AIDS-related deaths, 99% of which occur in developing countries. [10]–[12] Many TB suspects delay in seeking care due to HIV-associated stigma. [13]This not only increases the infectious pool within the community but delays initiation of effective chemotherapy. While TB disease can occur at any CD4 cell count, this risk increases as the immune system deteriorates. Similarly the likelihood of TB presenting in atypical and disseminated forms increases the more immune suppressed the individual becomes. [14], [15] Another important relationship between the two diseases is the difficulty in diagnosing TB in HIV-infected patients. Alteration in the clinical and radiographic presentation of TB and reliance on sputum smear microscopy and chest radiography whose diagnostic accuracy is substantially impaired in those with HIV co-infection make diagnosis difficult. [16]–[19] While these challenges might be surmounted by newer diagnostic technologies (e.g. Xpert MTB/RIF), these are not widely available in resource constrained settings such as sub Saharan Africa.

In sub Saharan Africa, antiretroviral treatment (ART) has improved survival and markedly reduced the incidence of new opportunistic infections (OI) including TB. [20], [21] Importantly, recently published randomized clinical trials (ACTG A5221/STRIDE and CAMELIA) have shown a mortality benefit in initiating ART early during TB treatment in HIV-infected patients. [22], [23] Whether the benefits of ART are similar between HIV-infected patients with or without TB, however, still remains unclear. Simultaneous treatment of TB and HIV is challenging due to high pill burden, medications side effects, potential drug interactions and the possibility of the Immune Reconstitution Inflammatory Syndrome (IRIS). Therefore, treatment outcomes may be adversely impacted. In Uganda, HIV-infected patients receiving ART had more subsequent OI and higher death rates if they were co-infected with TB at the time ART was initiated. [24] But in Malawi, patients with TB at ART initiation were found to have better survival rates and reduced incidence of new OI than those without TB [25].

Given the above contradictory findings, we analyzed data from a large cohort of HIV-infected patients who initiated ART at the Academic Model Providing Access to Healthcare (AMPATH) Partnership in western Kenya to determine the long-term differences in treatment outcomes between HIV-infected patients with and without TB disease at the time of ART initiation.

## Methods

### Study Site

Moi University School of Medicine (MUSoM), Moi Teaching and Referral Hospital (MTRH) and a collaboration of North American universities led by Indiana University School of Medicine (IUSM) direct AMPATH. [26] Established in November 2001 at MTRH in Eldoret, Kenya, AMPATH currently has HIV treatment, training and research programs in 80 Ministry of Health facilities spread throughout western Kenya. As of December 2007, when the data was extracted for this study, 64,008 HIV-infected adults and children had been enrolled. Of these, 49,172 were actively on follow-up, 23,437 (48%) of whom were on ART.

### TB and HIV Care

HIV and TB care are integrated. All patients attending TB clinic are tested for HIV under the Provider Initiated Testing and Counseling (PITC) program [27] and all HIV-infected patients are screened for active TB. Diagnosis of TB is based on guidelines from the Division of Leprosy, Tuberculosis and Lung Diseases of the Kenya Ministry of Public Health and Sanitation (adopted from the WHO). [28], [29] The TB diagnostic criteria include: 1) clinical presentation consistent with TB (cough for more than 2 weeks, unexplained weight loss, night sweats); 2) suggestive radiological findings (chest radiograph infiltrates consistent with TB, pleural effusion) and; 3) laboratory mycobacteriology (sputum smears, culture and tissue histology). During the period of study, TB treatment was conducted in 2 phases: an initial 2-month intensive phase with 4 anti-TB medicines (rifampicin, isoniazid, pyrazinamide and ethambutol) followed by a 6-month continuation phase with 2 anti-TB medicines (isoniazid and ethambutol).

All HIV-infected patients underwent a standard series of clinical, laboratory and radiological assessments and were assigned a WHO stage. ART was initiated in accordance with the national guidelines for Kenya at the time (CD4 cell count <200/ml, or being in WHO stage IV, or being in WHO stage III with CD4 cell count <350/ml). [30] The standard first-line ART regimen used was stavudine (or zidovudine) in combination with lamivudine and nevirapine. Efavirenz was substituted for nevirapine for patients on rifampicin. Co-trimoxazole preventative therapy was given to patients with CD4 cell counts <200/ml and to all patients diagnosed with TB. A nine-month course of isoniazid preventative therapy (IPT) was administered to patients without prior TB treatment and no clinical or radiological evidence of TB disease. For HIV-infected patients on anti-TB medication, timing of ART initiation was based on baseline CD4 cell count as per National ART guidelines: within 2 weeks from start of TB treatment for CD4 count <50/ml; within 4 weeks for CD4 cell count between 50 and 199/ml; and after completion of intensive phase anti-TB treatment for CD4 count ≥200/ml. However, clinicians were at liberty to initiate ART for an individual patient when they felt it was appropriate (e.g., the patient can tolerate additional medications).

Due to cost, viral loads are not routinely collected as part of care in this program. However, a viral load is drawn if a patient has clinical (worsening WHO stage) or immunologic evidence of ART failure (>50% drop in CD4 cell count, reduction in CD4 count below baseline 6 months after ART initiation or non-increment of CD4 count beyond 100 cells/ml after 1 year of ART). A viral load result of >10,000 copies/ml is considered definite ART failure while 1,000–10,000 copies/ml is considered probable failure.

Adherence to ART was measured using the patient’s report of the number of pills taken within the previous week (‘During the last 7 days, how many of his/her pills did the patient take?’) Perfect adherence to ART’ is defined as patient responding ‘All’ every time and ‘Non-Perfect adherence to ART’ if the patient ever answers ‘Most’, ‘Half’, ‘Few’, or ‘None. Information on hospitalization and death was received passively (as reported by relatives) and sought actively (from hospital surveillance and outreach team reports). Patients were considered Lost-To-Follow-Up (LFTU) if more than 3 months elapsed since their last visit while on ART, or more than 6 months if not on ART.

All patient data were recorded on standardized structured clinic encounter forms and later transcribed into the AMPATH Medical Record System (AMRS), [31], [32] a comprehensive electronic medical information management system using OpenMRS as its platform.

### Study Design

The MUSoM/MTRH Institutional Review and Ethics Committee (IREC) and the IUSM Institutional Review Board (IRB) approved the study. All IRBs waived patient informed consent for this project since the data used had no identifiers.

This retrospective cohort study utilized de-identified data from records of all ART-naïve adult, non-pregnant patients >18 years of age, who initiated ART between March 2004 and November 2007. Our working hypothesis was that HIV-infected patients initiating ART have worse clinical and immunological outcomes if they have TB at the time ART is initiated.

Patients were considered to have TB if evidence of TB treatment was recorded in the AMRS within a 10-month period beginning 8 months prior to initiation of ART and ending 2 months after. The 8-month period prior to ART initiation covered the TB treatment duration (2 months intensive phase and 6 months continuation phase) in Kenya during the study period (meaning patients have TB disease during that time). The 2-month period after ART initiation addresses cases of indolent TB, which may become unmasked as a result of IRIS.

While patient care is provided by clinical officers (physician assistants) and physicians, who can both record diagnoses in the patient record, we relied on treatment information to confirm TB diagnosis in the electronic medical record. In this analysis, all cases of TB are grouped together, irrespective of type of infection (e.g. milliary) and affected site (e.g. pulmonary, meningeal and pleural).

The primary clinical outcomes we assessed were mortality, AIDS-defining events (ADEs), first-line ART regimen failure, weight changes and immune system reconstitution (CD4 cell response) following ART initiation in both the TB and non-TB patients. ADEs were defined as any condition(s) listed as part of the WHO Stage IV criteria. [33] They were considered to have been present at baseline if they were diagnosed within 3 months prior to or up to 2 months after ART initiation. On the other hand, an ADE was considered to be incident if it occurred more than 2 months after initiation of ART. As with TB, this window was utilized to account for pre-existing conditions (present and likely to be active before ART initiation) and indolent infections that become manifest following immune improvement after ART initiation.

### Data Analysis

For purposes of this analysis, patients were categorized into 3 groups based on their CD4 cell count at ART initiation: 1) CD4<50 cells/ml; 2) CD4 50–199 cells/ml; and 3) CD4≥200 cells/ml. Responses to ART were compared between patients with TB and those without. Descriptive statistics were generated for all factors analyzed. While p-values <0.05 are considered statistically significant, due to the size of the cohort and the small p-values generated by even clinically meaningless differences, emphasis is placed on point estimates and 95% confidence intervals in describing differences between patient subgroups.

The effect of TB at the time of ART initiation on the occurrence of death, subsequent ADE, and LTFU rates was investigated through Cox Regression (Proportional Hazards) models, which were adjusted for baseline CD4 count category (defined above). For the mortality analysis, patients who remained alive until the end of follow-up or who were LTFU were censored on the date of their last visit. For the analysis of LTFU rates, subjects who remained on observation were censored at their last visit date, and those who were known to have died were censored on their date of death. For incident ADE, subjects who did not develop a new OI prior to November 2007 or who were lost or died without a known new infection, were censored at the last date they were known to be alive. These analyses were performed with the SAS system version 9.2 (SAS Institute, Cary, NC).

The impact of TB on CD4 cell count changes after ART initiation was assessed by using a piece-wise linear model of the square-root CD4 counts recorded at baseline (start of ART) and during follow-up. [34] Specifically, the early period after initiation of ART was modeled with a linear segment with a steep slope, while the later period was modeled according to a line segment with a less steep slope. Average (population-level) effects of active TB were tested as well as interactions of CD4 change and TB by use of generalized equation models (GEE). We searched through a number of candidate time points (weeks 1–26 after ART initiation) to estimate the temporal location of the changepoint. Interaction effects between TB group and time were also included in both phases. A negative interaction effect is associated with widening of TB-associated lags in CD4 counts over time, while a positive interaction implies a convergence (catching up) of TB subjects with their non-TB counterparts.

Weight changes post ART initiation were assessed by non-linear mixed models. We used a model of exponential weight increase, which prescribes that weight rises to a maximum (asymptote). The use of this asymptotic weight increase protects against the possibility that weight will be predicted by the statistical model as increasing past a maximum point, a biologically implausible situation. The model (in its most simple form) of the weight *Y_ij_* for subject *i* at visit *j* is given by the following equation:
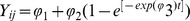



The first part of the model *φ*_1_ is the estimate of the weight at ART start, while the sum of the first and second part *φ*_1_+*φ_2_* is the estimate of the final (“asymptotic”) weight (when the exponential term becomes zero). The term *φ*_3_ in the exponential component (1-*e* ^[−exp(*φ*^3^)]^) governs how quickly the final weight (asymptote) is reached. We also included subject-specific (random) effects for the baseline weight. We adjusted our analyses for gender and baseline CD4 grouping as factors possibly associated with weight at baseline or with weight changes after ART initiation by including them as factors in the *φ*_1_ and *φ*_2_ parts respectively. We also assessed the impact of gender and baseline CD4 count on the speed of weight gain by including them as factors in *φ*_3_. These analyses were performed with the R language nlme package. [35], [36].

## Results

Between March 2004 and November 2007, 21,242 patients (62% females) fulfilling the study inclusion criteria initiated ART. Of these patients, 5,186 (24%) had active TB at ART initiation as defined in Methods.

### Baseline Characteristics

At the time of ART initiation, patients with TB as compared with those without TB: were of similar age; had a higher proportion of men; were more likely to be treated at an urban clinic; were significantly more immunosuppressed (had lower CD4 cell counts); had lower weight and BMI; and had a higher proportion of patients with one or more ADE. Adherence levels were comparable in both groups. Summaries of these results are given in Table 1.

**Table 1:** Baseline social, demographic and clinical characteristics of patients on ART in an ambulatory HIV care program in western Kenya, by TB status.

Baseline Characteristics	TB/HIV co-infectedpatients on ART N = 5,186	HIV-infected patientson ART N = 16,056	Absolute or % Difference (95% CIof difference^1^)
Age (years)			
Median (IQR)	37.0 (31.4, 43.4)	37.7 (31.6, 44.5)	0.70 (0.414, 1.000)
Male Gender	2,406 (46.4%)	5,600 (34.9%)	0.115 (0.010, 0.131)
	N = 4,927	N = 15,066	
Employed Outside Home	1,188 (24.1%)	3,258 (21.6%)	0.025 (0.011, 0.039)
Post-Primary (>8 years) Education	N = 4,635	N = 14,040	
	2,035 (43.9%)	5,677 (40.4%)	0.035 (0.018, 0.051)
Clinic Location			
Urban	2,724 (52.5%)	7,676 (47.8%)	0.047 (0.032, 0.063)
CD4 count (cells/ml) at ART start	N = 3,586	N = 12,673	
Median (IQR)	77 (29, 146)	109 (47, 175)	32.5 (25.85, 33.51)
CD4 count (cells/ml) at ART start	N = 3,586	N = 12,673	
0–49	1,330 (37.1%)	3,292 (26.0%)	0.111 (0.081, 0.141)^2^
50–199	1,810 (50.5%)	7,284 (57.5%)	−0.070 (−0.096, −0.044)
≥200	446 (12.4%)	2,097 (16.6%)	−0.041 (−0.076, −0.007)
Weight (Kg) at ART start	N = 5,285	N = 15,867	
Median (IQR)	52.5 (46, 59)	55 (48.5, 62)	2.5 (2.374, 3.031)
BMI	N = 4,579	N = 14,241	
Median (IQR)	18.7 (16.9, 20.7)	19.8 (17.8, 22.1)	1.1 (1.123, 1.341)
ADE^3^ (overall)	736 (14.2%)	1,317 (8.2%)	
CD4 (cells/ml) at ART start	N = 3,586	N = 12,673	0.06 (0.049, 0.070)
0–49	244 (18.4%)	415 (12.6%)	1.275 (1.027, 1.581)
50–199	237 (13.1%)	439 (6.1%)	1.100 (0.886, 1.364)
≥200	55 (12.3%)	194 (9.3%)	0.503 (0.359, 0.698)
Perfect Adherence to ART	N = 4,838	N = 14,777	
	3,852 (79.6%)	11,590 (78.4%)	0.012 (−0.001, 0.025)

1 Difference between proportions (categorical variables) or means (continuous variables) of TB versus non-TB group.

2 Difference in the proportion of subjects with CD4<50 cells/ml in the TB versus the non-TB group.

3 Patients presenting with an AIDS-defining event (other than extra-pulmonary TB) 3 months prior and 2 months after ART initiation (some CD4 data are missing).

### Survival, LTFU, Incident ADE and First-line ART Failure

**Figure 1:**
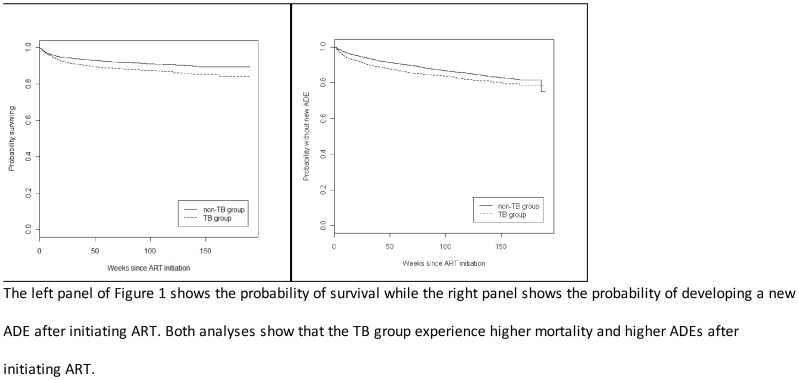
Kaplan-Meier curve of mortality (left) and new AIDS Defining Events (right) in the TB group (dashed line) versus the non-TB group (solid line).

As shown in Table 2, after adjusting for CD4 count at ART initiation, patients with TB disease had a 32% increase in adjusted mortality rate, were 31% more likely to experience a new ADE (both statistically significant) and 7% more likely to be lost to follow-up (not statistically significant). The rates of first-line regimen failure were comparable in the two groups. A pictorial presentation of these results is shown in Figure 1 and in Figures S1 and S2.

**Table 2:** Estimated hazard ratios for death, Loss To Follow-Up, incident AIDS Defining Event (ADE) and first-line ART failure for patients with TB/HIV initiating ART compared to non-TB co-infected patients.

	Unadjusted Hazard Ratio	Adjusted^1^ Hazard Ratio
Time from start of ART to …	(95% CI)	(95% CI)
Death	1.456 (1.304–1.626)	1.319 (1.180–1.474)
Loss-to-follow-up	1.124 (1.045–1.210)	1.068 (0.991–1.150)
Incident ADE^2^	1.395 (1.262–1.541)	1.313 (1.187–1.453)
Start of second-line ART^3^	1.048 (0.841–1.305)	0.989 (0.793–1.234)

1 Adjusted according to CD4 count at ART initiation.

2 Including extra-pulmonary TB.

3 Surrogate marker for 1^st^ Line ART Failure.

### CD4 Cell Response after ART Initiation

During the early phase, patients belonging to the two lowest baseline CD4 count groups experienced proportionally larger CD4 increases compared to patients in the highest group. This pattern continued past the sixth week post-ART initiation with these two groups continuing to experience higher relative CD4 increases compared to the highest baseline CD4 group. However, in absolute terms, even after one year, the differences in CD4 counts were large in all three groups (Table 3).

**Table 3:** Results from the piece-wise linear model for CD4 response after ART initiation.

Group	Baseline	Six weeks after ART	One year after ART
	CD4 count (95% CI^1^)	CD4 count (95% CI)	CD4 count (95% CI)
TB group (overall)<50 cells/ml50–199 cells/ml≥200 cells/ml	80 (75–86)17 (15–19)109 (100–119)297 (250–348)	200 (162–242)118 (91–149)232 (184–286)375 (267–501)	251 (210–295)172 (138–211)279 (230–333)414 (315–528)
Non-TB group (overall)<50 cells/ml50–199 cells/ml≥200 cells/ml	107 (101–113)21 (18–23)118 (112–123)311 (264–362)	221 (197–245)120 (96–146)234 (205–266)377 (291–475)	269 (246–293)173 (145–203)280 (251–310)415 (336–502)

1 Confidence intervals were derived based on the delta method of approximation, using estimates of the variance of the parameters produced by the GEE model. Note that the overall differences in CD4 counts present in the TB versus non-TB groups, are not evident among the three CD4 groupings suggesting that differences in CD4 response are a function of higher rates of baseline immunosuppression among TB patients rather than an independent TB-associated effect.

Overall CD4 levels at six weeks (200 versus 221 cells/µl) and one year (251 versus 269 cells/µl) were lower in the active TB group versus those without (Table 3). Once considered within each CD4 count category however (<50, 50–199 and ≥200 cells/µl) the six-week and one-year levels are virtually identical in the two groups. This may mean that the lower overall level seen in the active TB group at six weeks and one year is the result of the disproportionate representation of active TB patients in the lower CD4 strata.

### Weight Gain after ART Initiation

#### Effect of TB and gender on weight gain

At baseline, active-TB patients weighed on average 3.3 kg less than patients without TB after adjusting for gender and CD4 count at baseline (treatment initiation). However, active-TB patients gained on average 2.3 kg *more* than non-TB infected patients after ART initiation. Thus, even though ART (and anti-TB treatment) restores almost 70% of the weight gap between patients with active TB and those without (in both genders), those with TB continued to slightly (albeit significantly in the statistical sense) lag in weight behind without TB disease. The overall mean weight at one year after ART initiation was 60.4 kg (95% confidence interval – CI: 60.3–60.6) in the active TB group and 61.2 (60.8–61.5) in the Non-TB group.

#### Effect of initial CD4 cell count on weight gain

Patients with CD4 50–199 cells/ml were on average 4.1 kg heavier than patients with CD4 count <50 cells/ml at the time of ART initiation. Patients with CD4≥200 cells/ml weighed 7.0 kg more than patients with CD4<50 cells/ml. However, patients with CD4 50–199 cells/ml gained 3.9 kg *less* than individuals with CD4<50 cell/ml, effectively erasing the lag in weight between the two groups that was present at the start of ART. Although patients with CD4≥200 cells/ml gained 5.5 kg *less* than patients with CD4<50 cells/ml, differences persisted between the highest CD4 group and the lower two strata.

## Discussion

HIV-infected patients with active TB had higher rates of mortality and experienced more incident ADEs despite the observed robust response to ART. There are a number of possible reasons why this is the case. Patients with TB were more likely to have lower CD4 counts at the time they started ART, compared with patients without TB. In addition, TB was associated with a higher incidence of ADEs at the start of ART in all three CD4 groups. TB was also associated with, overall, lower average weight among both men and women starting ART, a strong predictor of survival. [37], [38] Thus, the combination of low CD4 count and weight along with the higher incidence of ADEs was likely the main cause of the higher observed mortality.

We found TB disease to be associated with lower CD4 cell counts at ART initiation. While both TB and non-TB patients experienced almost similar CD4 increases after ART initiation, overall CD4 counts were still lower in the TB group, even after a year from ART initiation.

Weight was lower in the TB group within all baseline CD4 strata as compared to the non-TB group. These effects are also reflected in the way that patients regained weight after initiating ART. Our study findings indicate that patients with TB gain weight rapidly but lag slightly behind non-TB patients even after a year of ART. This observation is particularly evident in the group of patients with CD4 cell counts at or above 200/ml at baseline. The clinical meaning of about one-kilogram residual difference in weight (<2% of total weight) between the TB and non-TB groups is not clear. Wheeler and colleagues analyzed results from four trials and found that even a mild weight loss of 5% over a short period to be associated with increased mortality and OIs. [37] However, it is unknown whether a <2% lag in weight gain would be associated with increased rates of adverse clinical outcomes.

Contrary to our hypothesis that the increased pill load and likely increased side-effects of anti-TB and ART co-administration would adversely impact ART adherence, we found that self-reported drug adherence was similar between HIV-infected patients with TB disease and those without. The similar levels of adherence in the two study groups likely explain the strong response of weight and CD4 counts to ART observed in all patients as well as in the similar risk of first-line regimen failure in the two groups. These results are encouraging indeed given the reported higher rates of treatment failure among patients with low CD4 counts at the start of therapy [39].

Last, but not least, we found that disproportionately more males than females were affected by TB. Although the reasons for the higher prevalence of TB among men are complex, it may be due to our previously published finding that men tend to seek care later in the course of HIV infection compared to women and thus have generally lower CD4 counts and more advanced WHO stage than women [40].

### Strengths and Limitations of the Study

The main strength of this study is the very large cohort of subjects studied in a resource-constrained setting representative of the realities of HIV and TB care and treatment in sub-Saharan Africa, where the epicenter of the two epidemics is located. This study involved well over 20,000 patients starting therapy and close to 40,000 CD4 cell count and 250,000 weight observations. However, the background within which this study was carried out is also responsible for a number of shortfalls, which are related to weaknesses inherent in routine clinical data collected in this setting. Diagnosis of TB is problematic in an area where most clinics do not have TB culture capability. This limitation also impacted our ability to differentiate infection with mycobacterial from non-mycobacterial acid fast bacilli. All adherence measurements were based on self reports, which we know do not capture true adherence status for patients. Also, the program does not conduct routine plasma viral load testing for monitoring of ART. This means that there may have been many more patients with 1^st^ line failure than we reported.

Another weakness of this study is the differential levels of death and LTFU in the TB and non-TB groups. The likely bias generated from these factors may have resulted in an overestimation of the CD4 and weight response to ART among TB patients and an underestimation of CD4 and weight differences between the TB and non-TB groups, particularly during the first weeks after ART initiation when the hazard of death is highest. Such biases may also account for some of the convergence of CD4 and weight trajectories in the two groups seen in the longitudinal CD4 and weight analyses. Thus, the association of TB with worse long-term outcomes may be even more serious than the data suggest, particularly since LTFU rates were higher in the TB group. Given our previous data on LTFU in our clinical setting, [41], [42] we postulate that a greater proportion of deaths occurred in patients who where LTFU as compared to those retained in care. Therefore, the impact of TB on mortality may be even higher than observed. For these reasons, although significant biases exist in our data, their nature and likely direction suggest that TB may have serious short and long-term impacts on HIV-infected individuals. The presence of these biases is thus unlikely to materially affect the main conclusions of this study.

### Conclusion and Recommendations

HIV-infected patients with active TB initiating ART have higher mortality and incident ADE rates compared to those without TB. While HIV/TB co-infected patients are generally more immunosuppressed, have higher rates of ADEs and weigh less at ART initiation the differences in initial CD4 cell count do not fully account for the worse outcomes observed in the TB patients, particularly given the robust response to ART experienced by all patients. These data suggest, therefore, that TB, on its own, is associated with additional risk for poor clinical outcomes beyond what would be explained through the association of TB with suppressed immune function (evidenced by the higher rates of low CD4 counts observed among TB patients starting ART). Thus, even though interventions to enhance early identification and comprehensive treatment of TB in HIV-infected patients may have salutary effects on survival, because they will increase the number of TB patients who initiate ART at higher CD4 counts, this will only partially address the higher mortality associated with TB/HIV co-infection. Initiation of ART at higher CD4 counts as well as a concerted TB prophylaxis effort in HIV-infected individuals will be necessary to reduce TB-associated mortality in this patient population.

## Supporting Information

Figure S1 Kaplan-Meier curve of hazard (instantaneous risk) of death in the TB group (dashed line) versus the non-TB group (solid line).
(TIF)

Figure S2 Kaplan-Meier curve of time from ART initiation to first-line ART regimen failure in the TB group (dashed line) versus the non-TB group (solid line).
(TIF)
